# Chemical Composition, Nutritional Quality and Yield in *Lactuca aff. indica* Varieties Using Multivariate Analysis Techniques

**DOI:** 10.1021/acsomega.5c02517

**Published:** 2025-08-26

**Authors:** Ramon Ivo Soares Avelar, Marcelo Henrique Avelar Mendes, Betsy Carolina Muñoz de Páez, Ana Beatriz Silva Araújo, Luciane Vilela Resende, Elisangela Elena Nunes Carvalho, Vytória Piscitelli Cavalcanti, Cleiton Lourenço de Oliveira

**Affiliations:** † Departament of Agriculture, Federal University of Lavras, Lavras, Minas Gerais CEP 37203-202, Brazil; ‡ School of Agricultural Sciences of Lavras (ESAL), 67739 Federal University of Lavras, Lavras, Minas Gerais CEP 37203-202, Brazil; § Departament of Food Science, Federal University of Lavras, Trevo Rotatório Professor Edmir Sá Santos, Lavras, Minas Gerais CEP 37203-202, Brazil; ∥ Postgraduate Program in Medicinal, Aromatic and Condimentary Plants Federal University of Lavras, Trevo Rotatório Professor Edmir Sá Santos, Lavras, Minas Gerais CEP 37203-202, Brazil

## Abstract

Multivariate analysis techniques can be useful for analyzing data that seeks to separate food plant cultivars according to yield, leaf quality and nutritional value. Thus, we used, validated and compared principal component analysis (PCA), Kohonen’s organizable maps (SOM), a nonsupervised competitive learning artificial neural network formed by a grid of (artificial neurons) and multifactorial analysis to differentiate three cultivars of tree *lettuce (Lactuca aff indica*), an unconventional food plant (PANC). For the differentiation, physicochemical variables of the leaves were evaluated in order to determine their sensory quality (total titratable acidity, total soluble solids, pH), antioxidant properties (vitamin C and total phenolics), nutritional and mineral composition (centesimal composition and macro and microelements), and yield results indicate that the highest leaf yield occurred in the first cutting for the three varieties evaluated, the nutritional quality of the leaves increased progressively as the experiment progressed, reaching significantly higher values in the third cut for some macro and micronutrients: Ca (278.9 g kg^–1^), Mg (64.1 9 g kg^–1^), S (2.53 g kg^–1^), Fe (1.78 g kg^–1^), Mn (1.93 mg 100 g^–1^). Among the varieties evaluated, SG stood out for presenting the best antioxidant properties, due to the highest concentration of ascorbic acid (355.18 mg 100 g^–1^) and total phenolics (569.2 mg GAE 100 g^–1^) in the second cut, along with a significantly higher average of free radical scavenging (SRL = 17.4%) when compared to PP and PR with values of 14.0 and 9.1%, respectively. According to the validation of the multivariate methods, all were suitable for analyzing the data obtained. SOM and PCA showed similar results, but PCA needed three components to be able to explain 75% of the data in a three-dimensional graph, while SOM made it possible to more efficiently explore the tendency to group the samples into seven groups according to the similarities and differences in the variables evaluated. This is due to its ability to reduce the size of the data and maintain a true representation of the relevant properties of the input vectors, generating a two-dimensional map that allows the results to be easily visualized.

## Graphical abstract

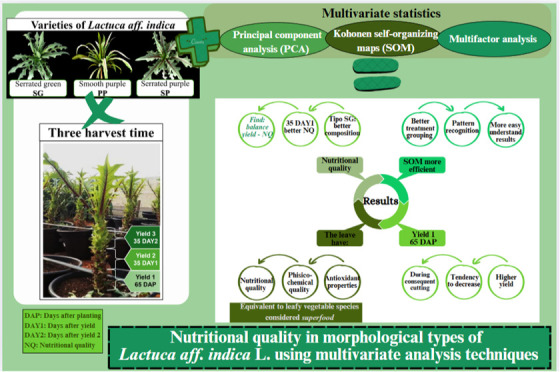

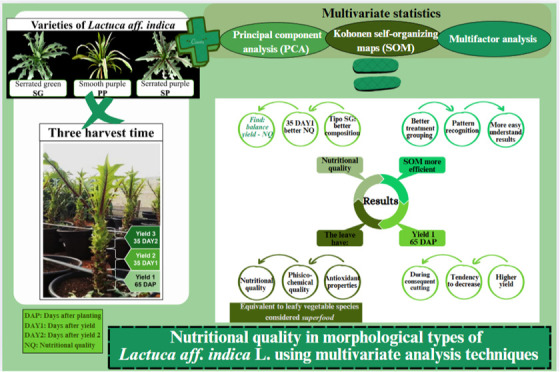

## 1 Introduction

There are various statistical methods and techniques for evaluating and comparing the differences between different species, genotypes, or plant varieties. In addition to univariate techniques, multivariate analysis techniques are an alternative for grouping or separating plants according to environment, management, analysis method and composition.^1–5^

Multivariate analysis is one of the most important areas of statistics, as it makes it possible to measure the response to phenomena in more than one variable, seeking to contemplate them in an integrated way, where the interrelationships are explored in maximum depth and the solutions to problems are more consistent and useful.^6,7^

Among the multivariate analyses that can be used for these purposes, principal component analysis (PCA) and Kohonen self-organizing maps (SOM) stand out. In this case, PCA has the advantage of allowing evaluation of the importance of each trait studied within the total variation available among the populations evaluated; and it can be used to discriminate chemical composition data obtained from plant food analyses.^8–10^ For their part, SOM is a type of artificial neural network trained through unsupervised competitive learning.^11^ It is a technique that has been unexplored in the field of agronomy, and more specifically in the differentiation of cultivars. This methodology performs nonlinear dimensionality reduction and good clustering, which is an excellent basis for good data visualization,^12^ facilitating the interpretation of results.

Bearing in mind that leafy green vegetables constitute a substantial component of the human diet and supply the majority of the nutrients, and bioactive compounds necessary for maintaining health and preventing disease,^13^ it is important to recognize that wild, edible, and underutilized plants—such as unconventional food plants (UFPs)—can be incorporated into the diet as a valuable source of nutritional supplementation. This practice has the potential to improve the socioeconomic conditions and health status of impoverished communities worldwide, particularly in developing countries.^14–17^ Therefore, it is essential to evaluate the food quality of these plant resources.

The quality characteristics of plant parts used as food result from interactions among diverse factors, especially genotype, environmental conditions, and crop practices^18–20^ Environmental factors, in particular, can induce stress responses in plants, potentially promoting or inhibiting specific physiological and biochemical pathways,^21,22^ thereby influencing the accumulation of nutrients and phytochemicals. Moreover, the inherent physiological diversity among plant tissues allows for considerable variation in the content of phytochemicals and antioxidant capacity,^23,24^ even within the same species, depending on morphological characteristics.

Preliminary studies evaluating the nutritional quality of UFPs have highlighted wild lettuce (*Lactuca aff. Indica*) as having considerable values of proteins and chemical composition, such as Ca, Zn, B, and Mn.^25,26^ Results of phytochemical and bioactive analyses of *Lactuca canadensis* showed the presence of fatty and organic acids, tocopherols, and phenolic compounds, and detected antioxidant and antibacterial activities.^27^ However, little information is found in the literature on differences in nutritional, chemical and antioxidant properties among the different varieties within the *L. aff. Indica*.

Furthermore, the optimal cutting time for maximum quality leafy vegetables is directly linked to consumer preference in each region, and this preference defines the maturity stage at which the product should be cutting, taking its preparation, destination, and storage potential into consideration.^28,29^ Additionally, consecutives cutting are common in leafy vegetables, and that can also affect the quality and yield of these species. Some authors affirm that this practice ensures higher yield per unit in terms of fresh and dry biomass, as well as gains in protein, carotenoid, and ascorbic acid content.^30–32^

In view of the above, the objective was to analyze and compare the nutritional quality of three varieties of tree *lettuce* (*Lactuca aff. indica*), in three successive cuttings, by means of PCA and SOM, in order to identify the differences in sensory quality, antioxidant properties, nutritional and mineral composition and yield, obtained from the application of PCA and SOM, in addition to comparing the results obtained by both analyses, determining which facilitates the understanding of the results

## 2 Materials and Methods

### 2.1 Experimental Site and Conditions

The study was conducted in a greenhouse at the geographical coordinates 21°14′ S, 45°00′ W, and 918 masl, from February to August 2017. The climate in the region according to the Köppen climate classification is Cwb, with a dry winter and rainy summer.^33^

The genetic material used was obtained from the germplasm collection of unconventional vegetables at UFLA (Figure 1). Sowing for seedling production was carried out in polyethylene plastic trays, seed trays with the following measurements: 34 cm long, 34 cm wide and 6 cm high; with 64 cells.

**Figure 1:**
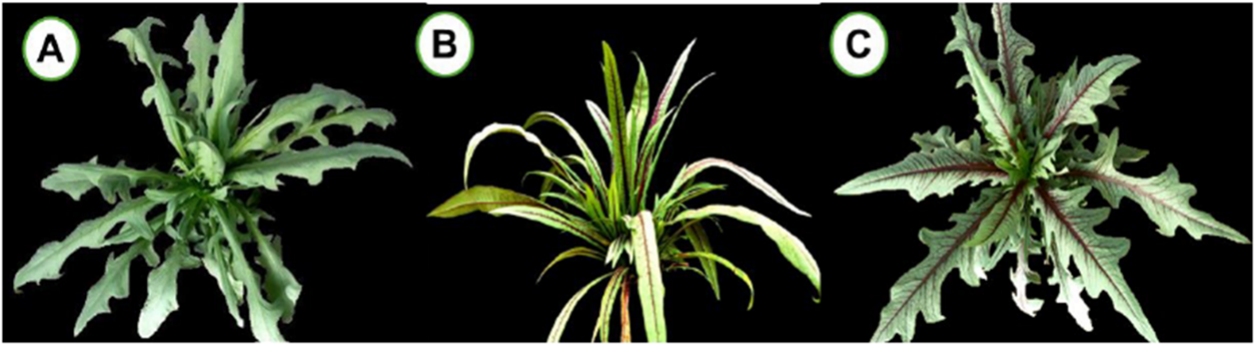
Varieties of Wild lettuce, differentiated by leaf shape and color: serrated green (A), smooth purple (B), and serrated purple (C).

In the seedling development phase, three leaf fertilizer applications were made using the commercial fertilizer Biofert [188.96 g L^–1^] (NPK 6–4–4, 0.5% Mg, 1% S, 0.02% B, 0.3% Cl, 0.02% Co, 0.05% Cu, 0.1% Fe, 0.05% Mn, 0.01% Mo, 0.1% Zn) every 7 days after emergence. Plants were irrigated using suspended and inverted microsprinklers, four times a day for 10 min, with a flow rate of 50L/h.

At 35 days after sowing, seedlings were planted in 5-L capacity polyethylene pots, with one plant per pot. In this phase, plants had three to four well-developed leaves.

Soil classified as a *Latossolo Vermelho Distroférrico* was used in the pots, and it had the characteristics shown in Table 1.

**Table 1:** Characteristics of the Soil Used as the Substrate (in the Tillable 0–20 cm Soil Layer)^a^

exchangeable Al	Ca^2+^	Mg^2+^	SB	CEC	Texture
cmol_c_ dm^–3^					
0.02	1.32	0.18	1.56	1.58	Clay

pH (H_2_O)	P (Mehlich)	K^+^	OM	V	M
	mg dm^–3^		g kg^–1^	%	
6.3	11.31	24.26	2.9	60.32	1.27

a SB: sum of bases, CEC: cation exchange capacity, OM: Organic matter, V: base saturation.

Fertilization at planting was based on soil analysis. For each pot, 4.5 L of soil and 0.7 L of organic compost were used. Nitrogen fertilization was carried out using ammonium sulfate [35.2 g L^–1^], applying 100 mL of the solution per pot at 21 days before cutting. Drip irrigation was used, with distribution in five rows spaced at 50 cm, with one emitter per plant. The plant spacing was 30 cm.

### 2.2 Experimental Design

The experiment was conducted in a completely randomized design in a 3 × 3 factorial arrangement. Factors consisted of three varieties of Wild lettuce which were identified as belonging to the species *Lactuca aff*. *indica* according to a recent publication of Avelar et al.^34^—the three varieties differentiated by leaf shape and color (serrated green—SG, smooth purple—PP, and serrated purple—SP), and three consecutive cuts of leaves [1, 2, and 3]. The first cut of leaves was carried out 65 days after planting, the second cut was 35 days after the first and the third cut 35 days after the second. The region on the plant where the leaves were cut did not recover, and cutting were characterized as follows: first cutting of leaves from the lower third, second cutting of leaves from the middle third, and third cutting from the upper third (Figure 2). Three replications of ten plants were used, for a total of 30 plants per varieties of Wild lettuce.

**Figure 2:**
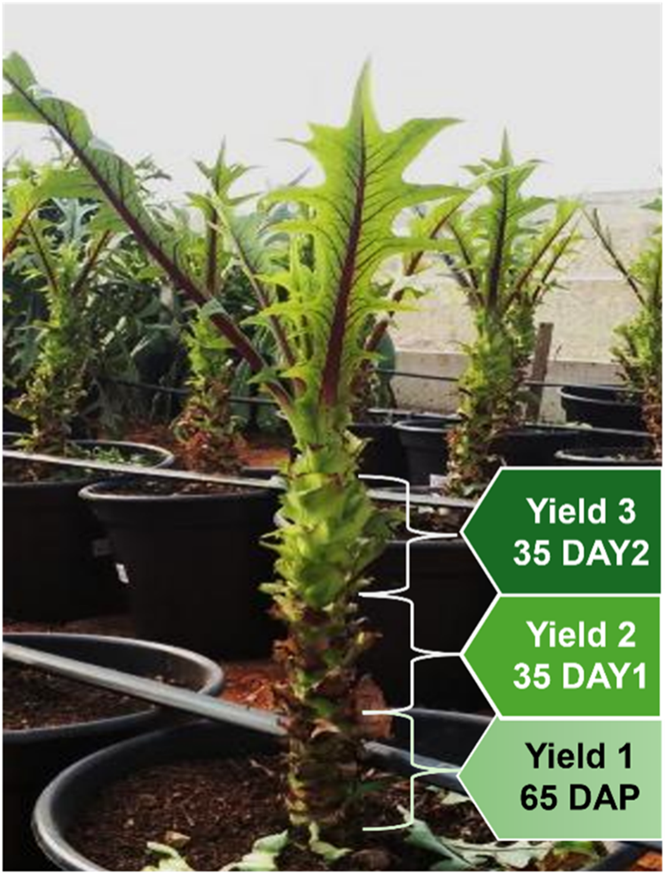
Plant regions where the cutting was carried out, depending on plant development.

### 2.3 Variables Analyzed

#### 2.3.1 Sampling and Quantification of Yield

At cutting, four leaves per plant were sampled for nutritional quality analysis, which amounted to around 300 g per repetition. All the mature leaves were then cut to quantify yield and only four newly formed leaves were kept ensuring retrovegetation of the plant for consecutive cuttings, as recommended by Brazil and Kinupp and Lorenzi.^35,36^

The plant material collected was divided into two parts. Of these, one was dried in an oven at 60 °C until constant weight to determine dry mass (DM) and moisture percentage (Mois). The dried samples were used for macroelements, microelements, ash and bromatological analyses. The other part, corresponding to the fresh plant material, was used immediately after collection to make alcoholic/aqueous extracts for antioxidant and sensory quality analyses.

#### 2.3.2 Physicochemical Analysis

Before each cut, the following assessments were carried out on the leaves of the plants: Chlorophyll content was determined by the relative chlorophyll index (CRI) using a chlorophyll meter, model SPAD-502. Coloration was determined using a Konica Minolta CR-400 colorimeter, calibrated according to the CIE system, measuring L*, a* and b* (illuminant D65); firmness was measured by the puncture test, using a Magness–Taylor penetrometer with a 3 mm diameter probe. Additionally, Firmness (Firm) was measured at the main vein of the leaf, by the puncture test using a Magness-Taylor penetrometer with a 3 mm diameter probe.

The samples were analyzed regarding proximate composition and bioactive compounds at the fruit and vegetable postharvest laboratory of the Department of Food Science, and the mineral analyses were conducted at the Nutrition Laboratory of the Department of Soil Science, both of UFLA.

The centesimal composition—including moisture, protein, lipids, fiber, carbohydrates, ash, and energetic value—was determined according to the official methodologies established by the Association of Official Analytical Chemists (AOAC).^37^ The pH was measured using a digital pH meter (Schott Handylab), and, together with the total soluble solids (TSS), it was analyzed following the procedures recommended by the Adolfo Lutz Institute.^38^ Titratable acidity was determined by titration with a 0.01 N sodium hydroxide (NaOH) solution, using phenolphthalein as an indicator, in accordance with the methodology described by AOAC.^37^ The results were expressed as a percentage (%) of citric acid equivalent present in the tree mushrooms. Based on the values obtained for titratable acidity and total soluble solids, the SS/TA ratio—an indicator related to flavor balance, commonly referred to as the *soluble solids to titratable acidity ratio*—was subsequently calculated.

Vitamin C was determined by the dinitrophenylhydrazine (2,4-DNPH) method according to (Strohecker and Henning, 1967); total phenolics were determined by the Folin-Ciocalteau method, as detailed by Waterhouse,^39^ using 50% methanol and 70% acetone for extraction. The antioxidant activity was determined using the free radical scavenging method (FRL), determining the inhibition of the *DPPH* (2,2-diphenyl-1-picrylhydrazyl) radical in the organic extracts obtained from the leaves according with Brand-Williams, adapted by Rufino.^40^

The determination of mineral content was performed on the extracts listed in Table 2, prepared according to the *Methodology for the Analysis of Elements in Plant Material* as described in the book *Evaluation of the Nutritional Status of Plants: Principles and Applications*.^41^ The quantifications were conducted using a UV–visible spectrophotometer (Bel, model MH5 V-M5), atomic absorption spectroscopy (PerkinElmer, model AAnalyst 400), and microdistillation, in accordance with the specific methodology designated for each element (Table 2).

**Table 2:** Extracts and Analytical Techniques Used for Mineral Quantification in Leaf Samples of Tree Lettuce^a^

type of element	mineral	extract/method	quantification
**Macro**	Ca and Mg	Nitro perchloric extract + addition of lanthanum	AAS
	N	Semimicro-Kjeldahl	microdistillation
	P	Metavanadate colorimetry	UVS
	K	Nitro perchloric extract	AAS
	S	Barium sulfate turbidimetry	UVS
**Micro**	Cu, Fe, Mn and Zn	Nitro perchloric extract	AAS
	B	Azomethine colorimetry H	AAS

a AAS: atomic absorption spectrophotometry; UVS: UV–visible spectrophotometry.

### 2.4 Statistical Analysis

#### 2.4.1 Principal Component Analysis and Kohonen Self-Organizing Maps

Prior to performing the multivariate analyses, the Box-Cox transformation, which maximizes the likelihood of the normal model,^42^ was applied to each variable when necessary (Appendix 2). The correlation matrix was constructed by calculating Pearson’s correlation coefficient between each pair of variables, which were then organized into a symmetric matrix. For these analyses, the data were centered and standardized, with a mean of 0 and a standard deviation of 1. Principal Component Analysis (PCA) was conducted to identify the variables contributing most significantly to the total variance, as proposed by Kassambara.^43^

The expected average contribution of a variable to a given principal component (PC) was calculated as 1/*n* × 100, where *n* represents the total number of variables. This criterion allowed the identification of the most influential variables in explaining data variability. The PCA was performed using the FactoMineR package version 2.4,^44^ and graphical outputs were generated with the factoextra package version 1.0 ^45^ in R software. The adequacy of the data for PCA was assessed prior to analysis.

The validity of PCA was first evaluated through Bartlett’s test of sphericity, which examines whether the correlation matrix significantly differs from an identity matrix—indicating the presence of meaningful correlations among the variables. If the correlation matrix approximates an identity matrix, this would suggest very weak correlations between variables (i.e., all correlation coefficients near zero). The cross-validation method was also applied to validate the PCA, using the PLS package^46^ do R software version 4.0.3.^47^ For this validation, resampling was carried out with 60% of the data as training and 40% as test.

Another multivariate technique applied in this study was Kohonen’s Self-Organizing Maps (SOM), used to classify samples into clusters based on the similarity of their properties. The analyses were performed using the SOM Toolbox 2.1 ^48^ in MATLAB R2015a, with appropriate adjustments to improve cluster formation and validation, employing the Davies-Bouldin and Silhouette indices. The Davies-Bouldin Index^49^ evaluates clustering quality by considering both the within-cluster dispersion and the separation between clusters, where lower values indicate better clustering, with zero as the theoretical optimum. Cluster cohesion was further assessed using the Silhouette Index,^50^ a method for interpreting and validating the consistency within data clusters.

#### 2.4.2 Factor Analysis

Factor analysis was conducted following the confirmation of data adequacy through Bartlett’s test of sphericity. In this method, the effects of factors and their interactions were analyzed using Analysis of Variance (ANOVA), and when significant effects were detected (*p* < 0.05), mean comparisons were performed using Tukey’s test at a 5% significance level. These statistical procedures were executed using the ExpDes.pt package^51^ in R software version 4.0.3 (R Core Team, 2020). The validity of the factor analysis models was additionally verified through visual inspection of histograms and assessment of residual normality.

## 3 Results

### 3.1 Principal Component Analysis and Self-Organizing Maps

In Bartlett’s test of sphericity, the *p*-value < 0.01 shows that the correlation matrix is not an identity matrix. It is appropriate to continue with PCA. Thus, the graph resulting from the PCA (Figure 3) shows that 75.7% of the variance in the experiment is explained by the first three components, reducing the size of the data from 28 variables to 3 components. In the image we can see the similarity between treatments G2 and G3, associated with AT, EnV, Prot, Ash and Lip, as well as Fe and S. It is also possible to see the proximity of treatments PP3 and SP3, associated with the macroelements Ca, Mg and P, and the microelements Cu and Mn. Similarly, it can be seen that treatments PP2 and SP2 are similar to each other and are associated with pH, AAc and Zn. In the graph, it is clear that the treatments that include the first collection (SG1, PP1 and SP1) were the most different. These treatments are possibly associated with Mois, K and Prod and TP respectively.

**Figure 3:**
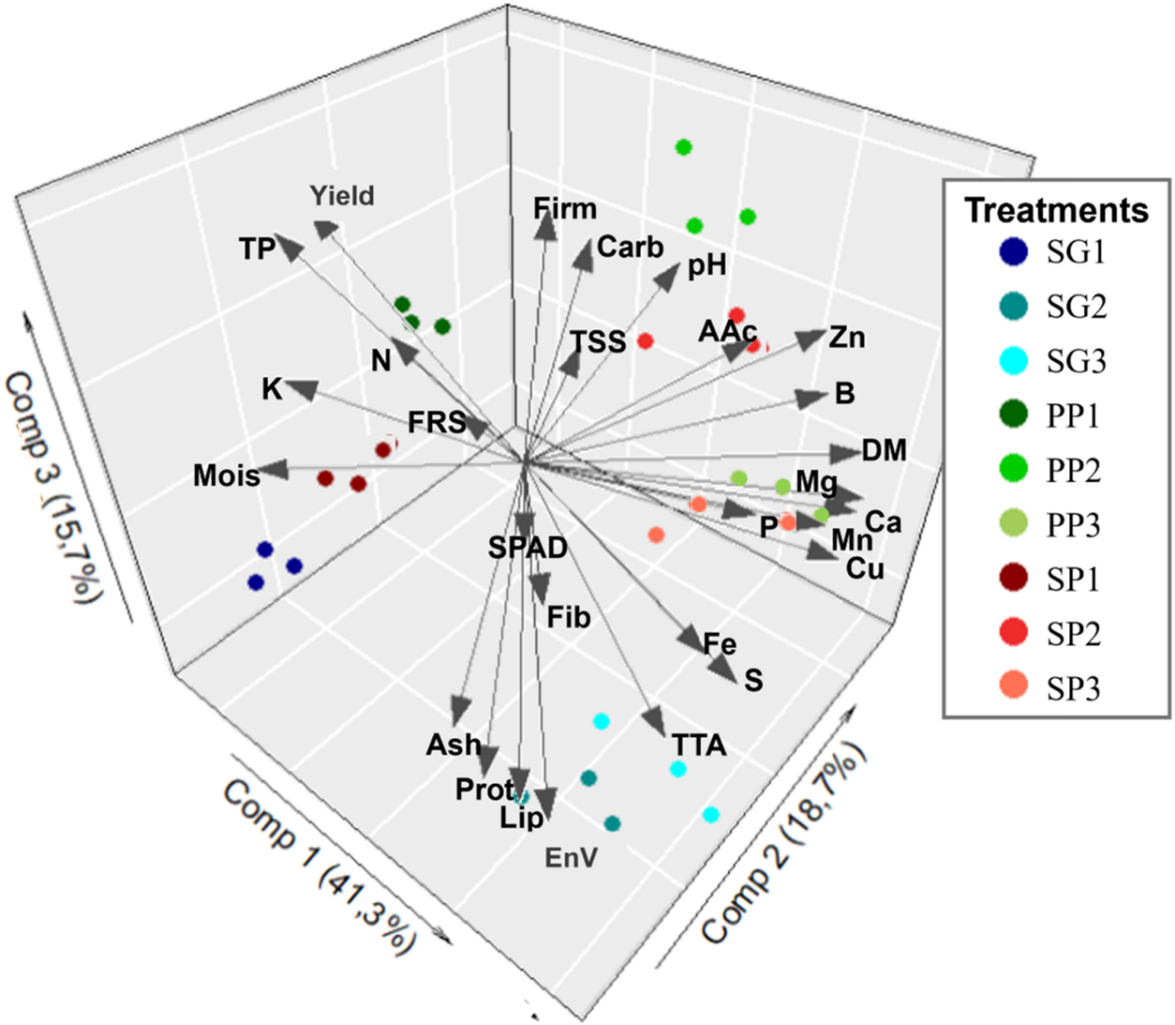
Biplot showing the results of the PCA. Arrows represent the variables studied and dots the treatments.

The cross-validation demonstrated what 3 components are sufficient to explain over 75% of the variability in the data.

As for the self-organizing maps, maps with different dimensions were generated in order to obtain the arrangement with the best cluster validation indices (Davies-Bouldin and Silhouette indices), along with the lowest measurement errors (quantification, topographic and combined). The network chosen in this study was a 2 × 21 hexagonal network with seven clusters, a Davies-Bouldin index of 0.5122 and a Silhouette index of 0.7698.

Each hexagon in the two-dimensional ANN/KSOM neural map represents a neuron, in which the treatments studied are grouped according to their similarities. Based on this assumption, it was possible to identify the division of the samples into seven groups (Figure 3A). The component maps of each analysis and the U matrix are shown in Figure 3B. The color scale indicates the distance between adjacent neurons and the variation in the results obtained in the analytical determinations can be seen by the color gradient of the bars located at the bottom of each map, with the exception of the U matrix.

Considering that the position occupied by a sample on the neural map (Figure 3A) corresponds to its same position on the component map (Figure 3B), it was possible to identify the main variables responsible for clustering and separation of the samples. Thus, first of all, we can observe the similarity between the SG2 and SG3 treatments in the cluster on the far left of the map, whose grouping was determined by the variables Prot, Lip, EnV, Ash, Fe, and S, as well as Ca, Mg, and B. For their part, the PP2 and SP2 treatments were also grouped in the cluster on the far right of the map, affected by DM, Ca, Mg, B, Cu, and Zn. The PP3 and SP3 treatments, even though in separate clusters, are adjacent and share their association with Mg and Lip, and they are well differentiated by Fe, Zn, and Mn.

The formation of the central clusters (samples from the first cut) was highly affected by the high values of yield, moisture, and SS, with N and TP standing out in SG1 and SP1; K in SP1; and Carb and FRS in PP1 (Figure 4).

**Figure 4:**
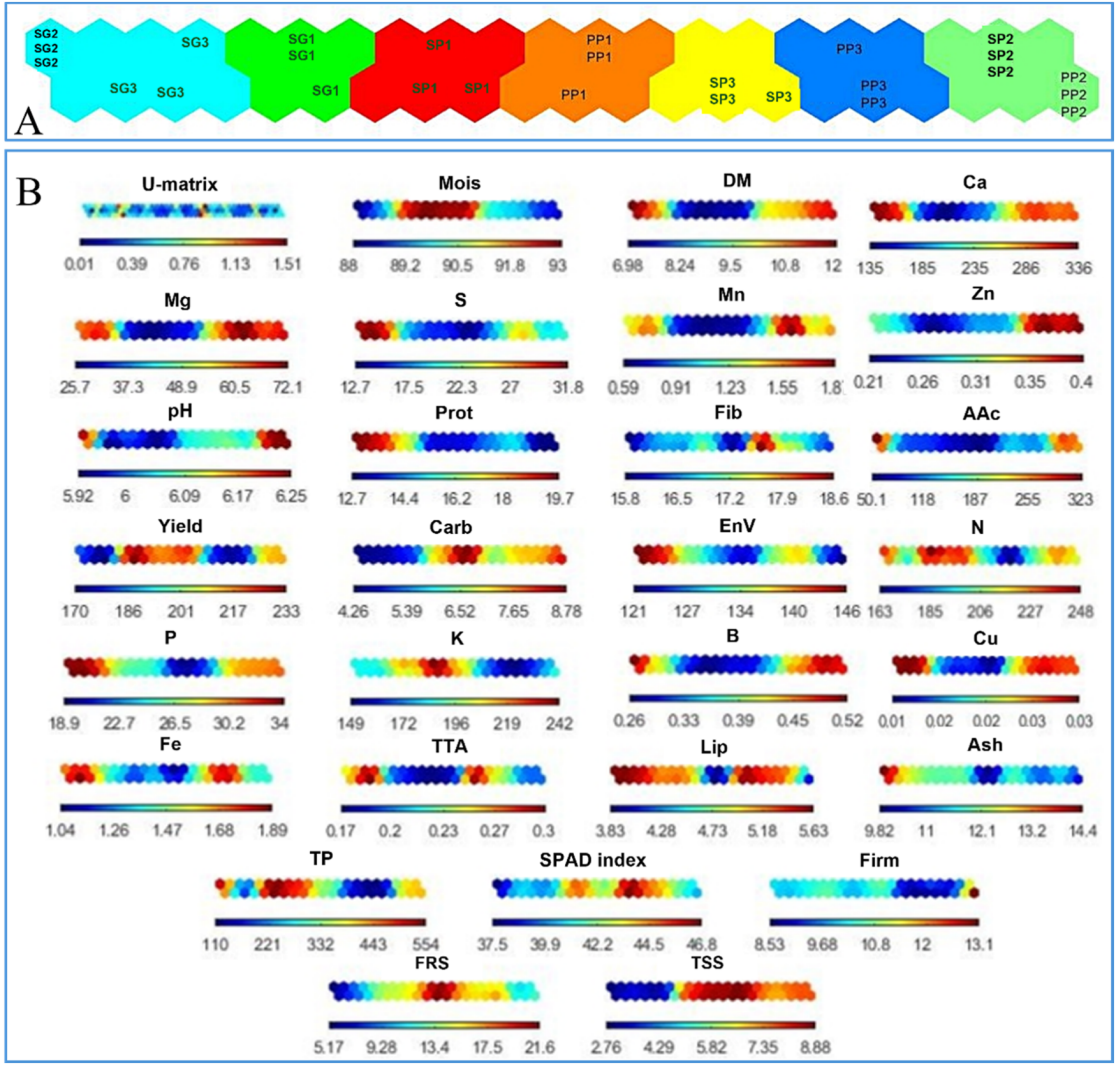
Two-dimensional neural cluster map showing the formation of three groups with their respective treatment and cutting times (A) and component maps and distance matrix (U-matrix) for data related to yield and to physicochemical analyses (firmness, moisture, soluble solids (SS), pH, titratable acidity, and chlorophyll), proximate composition (protein, ash, lipids, and fiber), minerals (N, P, K, Cu, Ca, Fe, Mg, Mn, S, B, and Zn), and antioxidant activity (total phenolics, ascorbic acid, and % FRS—DPPH) (B).

### 3.2 Factor Analysis

Significant interactions among the varieties of Wild lettuce and cutting times were observed for the Prod, Firm, TP, AAc, and Lip variables.

The absence of a significant cutting × variety interaction for a specific trait indicates that both the classification of the varieties and the magnitude of the differences between the varieties were similar over the different cutting times.

The three varieties of Wild lettuce evaluated had the highest yield in the first cutting (Figure 5A), with gradual decline up to the last cutting, which had the lowest values. The green morphological type stood out with the highest yield in the first cutting but was the lowest producing in the second and third cuttings. In the case of firmness (Figure 5B), the leaves of the PP variety stood out with increased firmness in the second cutting, unlike the other varieties, in which leaf firmness gradually declined over successive cuttings. This same PP variety of Wild lettuce showed an increase in the TP values in the leaves in the second cutting, but it was still below the SG variety, whose TP values in the leaves were highest over the time of the experiment (Figure 5C). For the three varieties evaluated, the peak production of ascorbic acid occurred in the second cutting (Figure 5D), and it was highest in the green morphological type, except in the third cutting, where its ascorbic acid value was below the values of the other two types evaluated. The contrary occurred for Lip, which was lowest in the second cutting for the three varieties evaluated; the SP type stood out, whose leaves showed the smallest differences in lipid content across the evaluations (Figure 5F).

**Figure 5:**
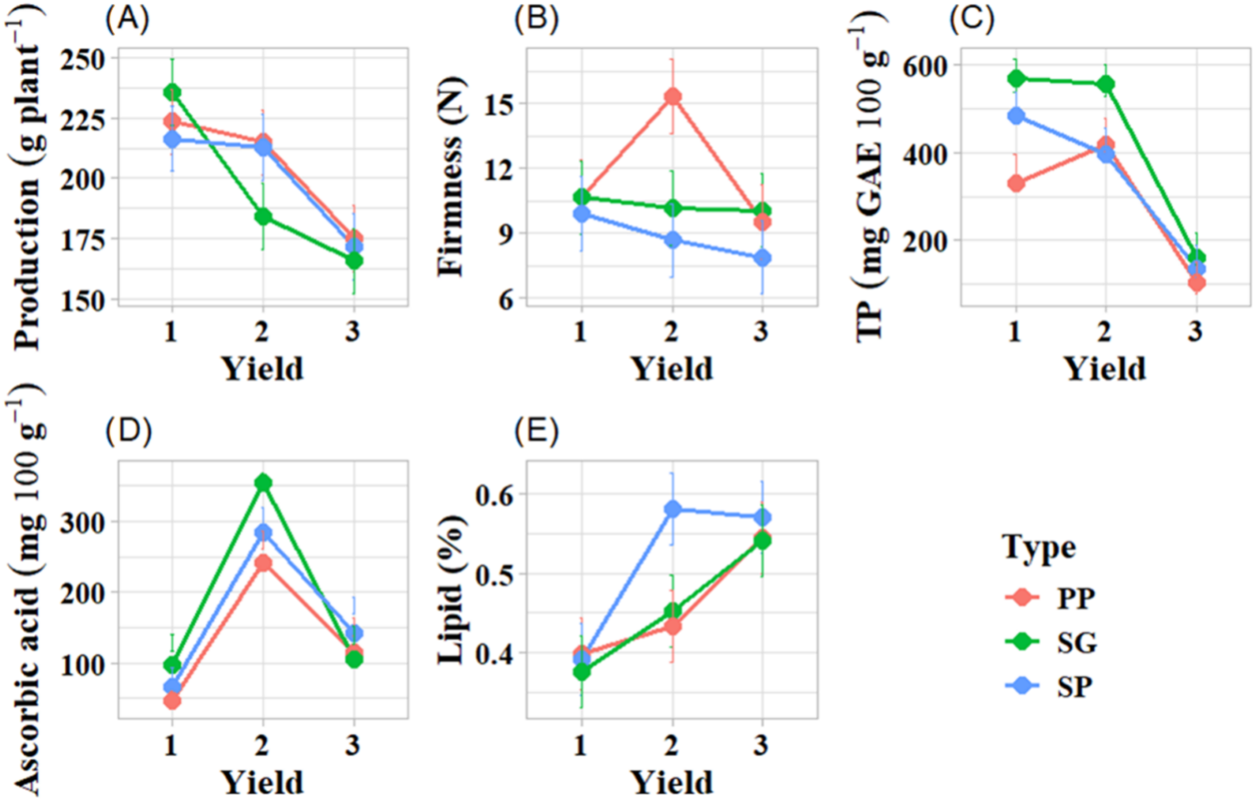
Positive interactions for the type and cutting factors in the following variables: (A) yield, (B) firmness, (C) total phenolics, (D) ascorbic acid, and (E) lipids. Varieties of Wild lettuce: serrated green (SG), plain purple (PP), serrated purple (SP). Cuttings: 1 (65 days after planting), 2 (100 days after planting), and 3 (135 days after planting). The dots on the graph represent the mean values for the treatments (variety: cutting combination), while the bars represent the confidence intervals (95%) indicating the uncertainty associated with the estimates of interactions between factors.

When we consider the chemical composition of the leaves, there was interaction between variety and cutting only for N and P. The SG and SP variety showed a decrease in N content over the evaluations, unlike PP, whose N values increased in the latter cuttings. Similarly, the values of P were higher for all the varieties in the second cutting, with SG standing out, as its values of P were significantly higher in all the evaluations (Figure 6).

**Figure 6:**
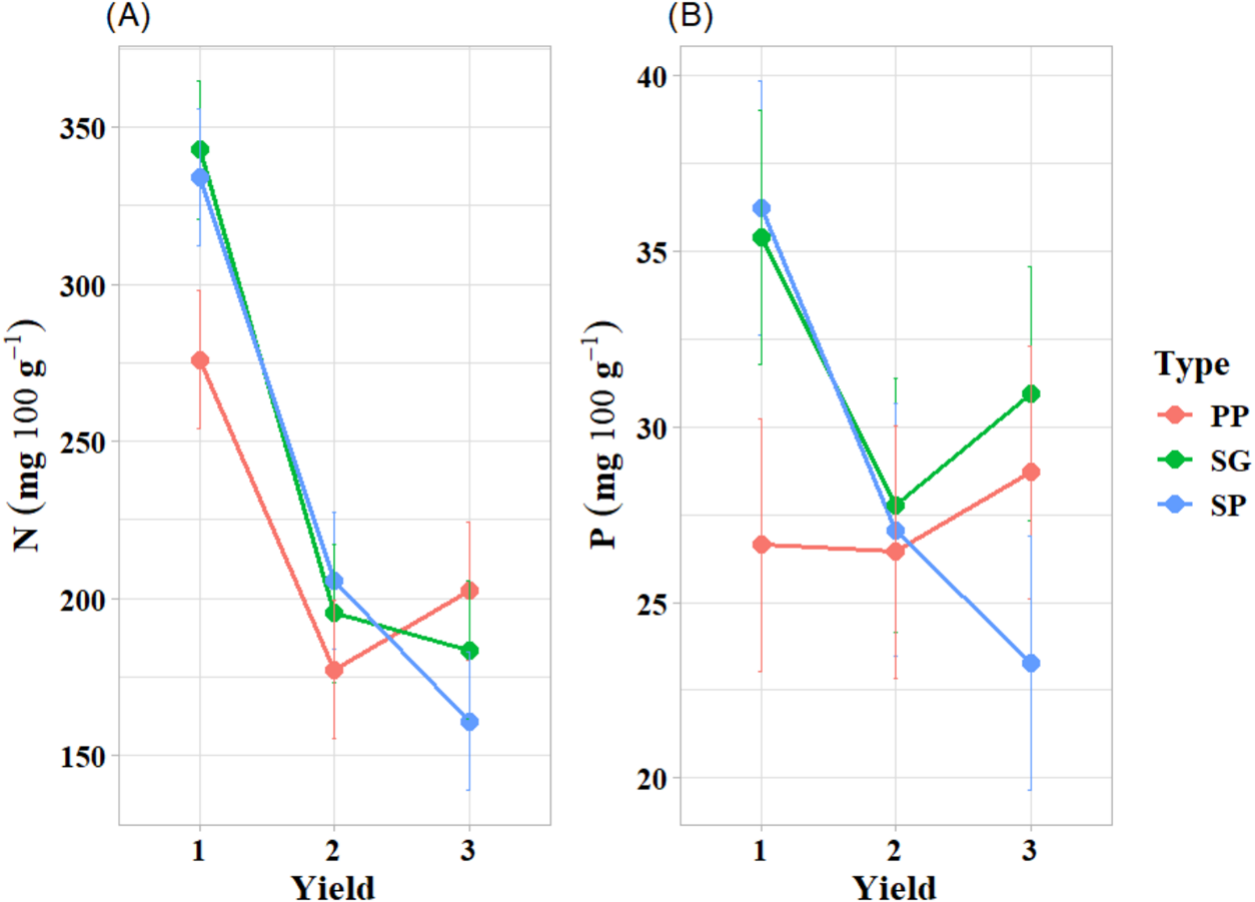
Effect of the significant interactions between the type of Wild lettuce and the cutting time factors on (A) nitrogen and (B) phosphorus. Varieties of Wild lettuce: serrated green (SG), plain purple (PP), serrated purple (SP). Cuttings: 1 (65 days after planting), 2 (100 days after planting), and 3 (135 days after planting). The dots on the graph represent the mean values for the treatments (variety: cutting combination), while the bars represent the confidence intervals (95%) indicating the uncertainty associated with the estimates of interactions between factors.

For the other variables evaluated in this study, the interaction between the factors was not significant. However, the cutting has a significant effect on yield and nutritional variables, as shown in Figure 7. The leaves gathered in the first cutting had significantly higher values for proteins (Figure 7H), ash (Figure 7J), energetic value (Figure 7L), and potassium (Figure 7N). Similarly, this first cutting showed the lowest values for dry matter (Figure 7A), total titratable acidity (Figure 7C), total soluble solids (Figure 7D), TTA/TSS ratio (Figure 7E), carbohydrates (Figure 7I), fiber (Figure 7K), antioxidant activity (Figure 7M), and the minerals Mg, S, Cu, Fe, Mn, B, and Zn (Figure 7O to 7V). The second cutting exhibited statistically higher values for dry matter (Figure 7A), pH (Figure 7B), TTA/TSS ratio (Figure 7E), carbohydrates (Figure 7I), and boron (Figure 7U). Meanwhile, the SPAD index (Figure 7F), fiber (Figure 7K), and Fe (Figure 7S) were significantly higher in the third cutting.

**Figure 7:**
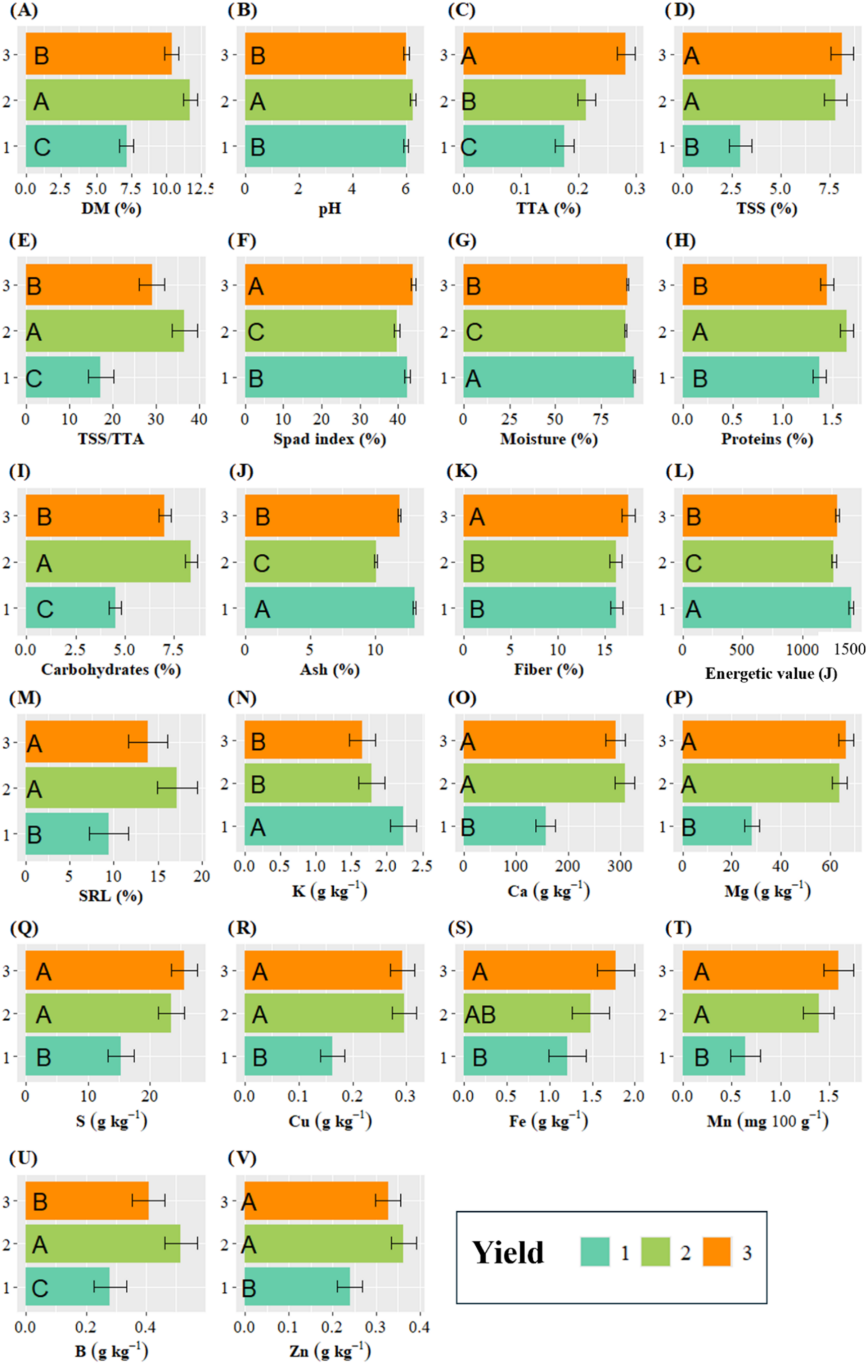
Effect of cutting time of Wild lettuce on (A) dry matter, (B) pH, (C) total titratable acidity, (D) total soluble solids, (E) TTA/TTS, (F) SPAD index, S (G) moisture, (H) proteins, (I) carbohydrates, (J) ash, (K) fiber, (L) energetic value, (M) DPPH free radical scavenging, (N) potassium, (O) calcium, (P) magnesium, (Q) sulfur, (R) copper, (S) iron, (T) manganese, (U) boron, (V) zinc. Cuttings: 1 (65 days after planting), 2 (100 days after planting), and 3 (135 days after planting). The error bars represent the confidence intervals, indicating the range of probable values where the true population means may be located, with a confidence level of 95%.

Furthermore, the morphological type of wild lettuce (*Lactuca aff. indica*) led to significant differences for seven variables (Figure 8). Thus, the SP type showed significantly lower values for carbohydrates (Figure 8B), Ca (Figure 8D), Mg (Figure 8E), and Mn (Figure 8G), and only showed significantly higher values for chlorophyll (Figure 8A) compared to the SG and PP varieties. The SG variety proved to be superior in S content (Figure 8F) in the leaves, while the PP type had leaves with significantly higher Mg (Figure 8E) and Mn (Figure 8G) values.

**Figure 8:**
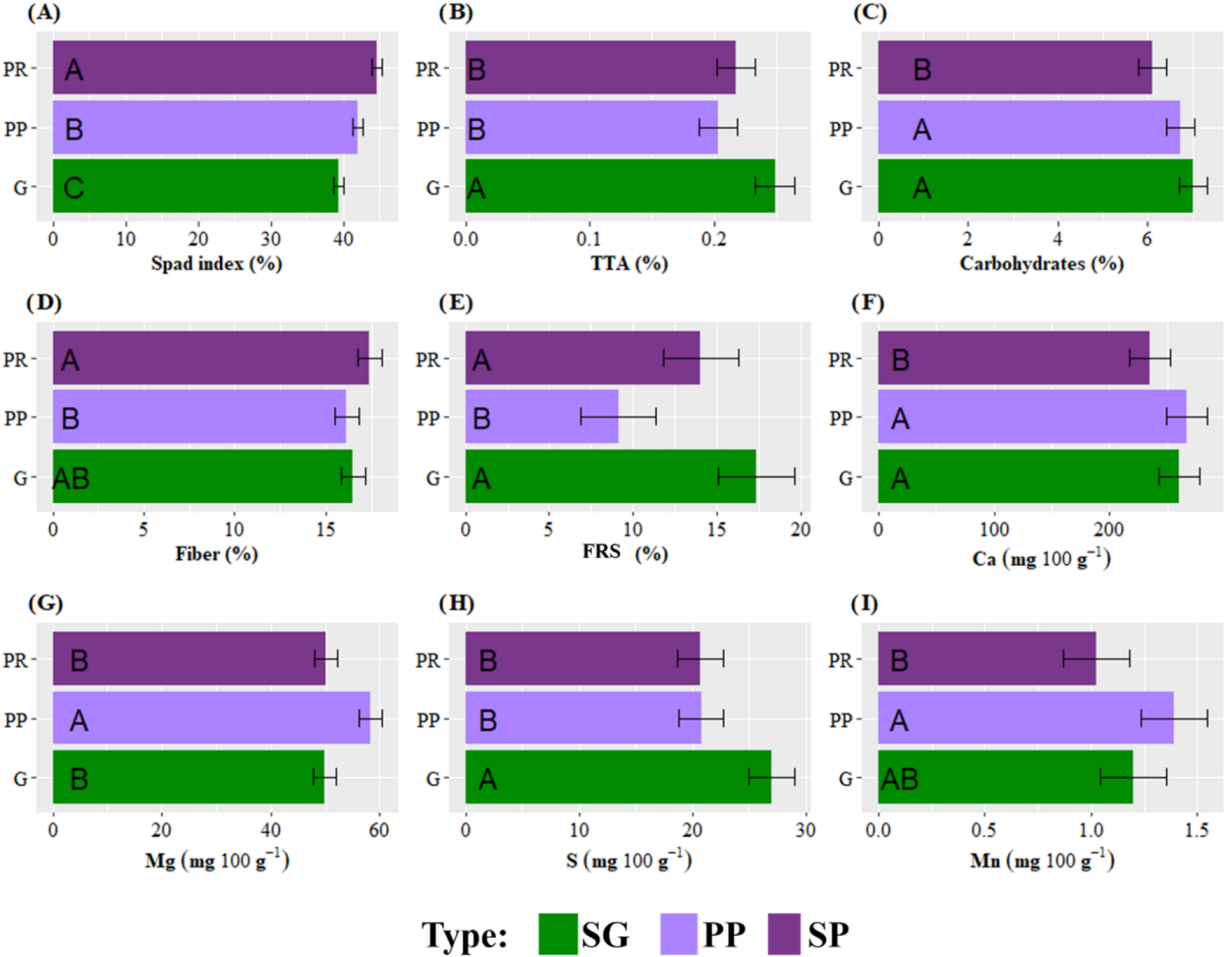
Response of the variables: (A) SPAD index, (B) Titratable Total Acidity (TTA), (C) Carbohydrates, (D) Fiber, (E) Free radical scavenging (FRS), (F) Calcium (Ca), (G) Magnesium (Mg), (H) Sulfur (S), and (I) Manganese (Mn), according to the type of Wild lettuce. Varieties of Wild lettuce: serrated green leaf (SG), plain purple leaf (PP), serrated purple leaf (SP). The error bars represent the confidence intervals, indicating the range of probable values where the true population means may be located, with a confidence level of 95%.

## 4 Discussion

### 4.1 Principal Component Analysis and Kohonen Self-Organizing Map

Principal Component Analysis (PCA) simplifies the complexity of high-dimensional data while preserving underlying trends and patterns. It achieves this by transforming the original data into a reduced set of dimensions, which serve as summaries of its main characteristics.^52^ Principal Component Regression (PCR) is a widely recognized least-squares estimator employed to determine the number of components that account for the greatest variability within the data,^53^ as well as to identify how the evaluated variables are grouped. Cross-validation (CV) is a very popular technique for model selection and model validation.^54^ When applied in combination, cross-validation and PCR enable the assessment of PCA’s stability. In this study, the Principal Component Regression (PCR) revealed that, after multiple resampling procedures, the variability explained by the first three components consistently ranged between 70 and 80%, with an average of 75.3%. Therefore, the number of components selected was deemed appropriate for the data set analyzed, and the resulting PCA effectively represented the interactions between treatments and variables. On other hand, the validation of SOM is done using cluster validation indices, with emphasis on the Davies-Bouldin index (DBI), which identifies well-separated clusters of clusters, and the silhouette index that calculates a measure of quality for each sample.^55^ These index, confirmed that the data were satisfactorily clustered (**Davies-Bouldin Index = 0.3122**), with strong cluster cohesion and consistency, as indicated by the **Silhouette Index = 0.7698**. These values demonstrate that, like PCA, the SOM was suitable for analyzing and representing the data set.

Although both multivariate methods proved statistically adequate for data analysis, the SOM (Figure 4) demonstrated superior performance in organizing multivariate data based on similarity, efficiently reducing data set dimensionality and facilitating interpretation of relationships when compared to PCA (Figure 3), as also observed by Martín-Fernández et al.^56^ According to these authors, this advantage arises because PCA projects objects onto a continuous output space, while SOM employs a regular grid of nodes onto which samples are projected. In many cases—including this study—PCA requires more than two axes to adequately capture data variance, and by design, PCA may produce misleading interpretations when group structures exist in the data.^57^ Similarly, in studies evaluating clustering efficiency, it was found that SOM yielded lower mean square errors in cross-validation and prediction, suggesting greater efficiency in variable clustering. In contrast, other authors observed that while PCA exhibited good discriminatory power when evaluating cocoa content in chocolate samples, SOM performed better as a complementary tool for pattern recognition.^58^

Considering that, in SOM analyses, similar colors indicate direct relationships between parameters,^12^ the SOM component plane (Figure 4B) revealed strong correlations between plant dry matter and nutrients such as Ca, Mg, S, B, Cu, as well as Vitamin C and titratable acidity (AT). The SOM results in this study indicate that one of the primary factors influencing the biogenic element content of *Lactuca aff. indica* varieties is leaf dry matter, given its directly proportional relationship with mineral concentrations—leaves with lower dry matter content presented lower nutrient concentrations, and vice versa. This finding was corroborated by the moisture content distribution, as the highest moisture values (represented in red on Figure 4B) coincided with the lowest nutrient concentrations, with the exception of nitrogen (N) and potassium (K). Similar trends were reported in studies examining nutrient-dry matter relationships in cauliflower florets, stems, and leaves, where linear increases in growth and dry matter accumulation were associated with higher nutrient contents. In that study, the descending order of nutrient accumulation was N > K > Ca > S > Mg > P > Fe > Mn > Zn > B > Cu.^123^

The SOM component plane provided a graphical representation of the relationship between each input variable and the evaluated *L. aff. indica* varieties (Figure 4A,B). According to the scale bars, higher values are represented by red and lower values by dark blue, consistent with the visualizations presented by Mamouni et al. and Ramli et al.^59,60^ Based on these interpretations, treatments G2 and G3 were grouped due to their higher contents of proteins, lipids, energetic value, and AT, alongside elevated concentrations of Ca, Mg, S, Cu, and P. However, the variables differentiating SG2 from SG3 included pH, Vitamin C, ash, N, B, and total phenolics (TP), which reached their highest levels in SG2. Comparable findings were reported in nutritional characterization studies of unconventional food plants from Angola, where species such as *Amaranthus hybridus*, *Bidens pilosa*, and *Galinsoga parviflora* exhibited similarly high protein and copper concentrations, comparable phosphorus and sulfur levels, and lower calcium and lipid contents.^61^

Likewise, treatments PP2 and SP2 clustered together due to their similar microelement concentrations, with high values of Mg, Cu, B, Zn, and Vitamin C, as well as the highest pH values. These two treatments differed primarily in firmness—highest in PP2—and lipid content, which was lowest in PP2, comparable only to PP1. In studies of Southeast Asian unconventional food plants, specifically freshwater macrophytes (*Eichhornia crassipes*, *Limnocharis flava*, and *Neptunia oleracea*), copper levels were similarly low to those observed in *L. aff. indica*, while Mg and Fe contents were lower, with the exception of *N. oleracea*, whose leaves had higher Fe concentrations.^62^

Conversely, treatments from the third cutting of PP and SP, which were positioned closely in both multivariate analyses, were characterized by lower total leaf yield, accompanied by the lowest TP values and the highest lipid contents. However, their separation into distinct SOM clusters was determined by the highest fiber content in SP3 and higher concentrations of S, energetic value, Fe, Mn, and B in PP3. The phenolic compound content in these lower-producing groups averaged approximately 105 mg GAE 100 g^–1^, consistent with levels reported for de M. Souza et al.^63^ in *C. prunifera*, and substantially lower than those recorded in SG1, where values exceeded 500 mg GAE 100 g^–1^. Considering the importance of total phenolics as antioxidant compounds, as emphasized by the authors, the nutritional differences among these groups are highly relevant.

Furthermore, data from the first cutting (SG1, PP1, and SP1) exhibited the greatest differences, with the lowest nutrient, vitamin C, pH, and AT values, but the highest yields and moisture contents. According to the SOM analysis (Figure 4), the differentiation of *L. aff. indica* varieties in the first cutting were primarily associated with higher production and protein content in SG1, higher potassium content in SP1, and greater carbohydrate but lower lipid content in PP1. Unlike the results of this study, previous research on PANCs (Unconventional Food Plants) has typically observed yield increases following successive cuttings. Notably, this final treatment (PP1) also exhibited the lowest nitrogen content in the first cutting. In African PANC studies, Houdegbe et al.^64^ tested successive cuttings of edible leaves at two- and three-week intervals, with the highest yields occurring 2 weeks after the first cut—through these yields did not surpass those of the initial cutting. In another investigation involving *Cleome gynandra*, also an African PANC, yield fluctuations between cuttings were influenced by the cutting method, with entire stem cuts resulting in higher yields compared to selective tender leaf cuttings. These findings underscore the importance of pioneering studies focusing on the morphogenic and structural development of this plant, as such insights may inform optimized cutting strategies to improve both yield and nutritional quality.^65^

Regarding leaf nutritional quality, it is well established that nutrient concentrations (including proteins and macro- and micronutrients) typically decrease in leaves after the first cut. However, in the present study, the highest concentrations of macro- and micronutrients, as well as antioxidant compounds, were found in the second and third cuttings, varying by morphological type. In this context, the SG type exhibited the highest concentrations of Ca, S, P, Cu, Fe, B, and energetic value, while the PP and SP types stood out for their higher Mg, Zn, and B contents. These differences likely reflect the effects of cutting-induced stress, plant ecophysiology, the interval between cuttings, and the fertilization practices adopted after cutting—as discussed in several studies.^31,65–67^

### 4.2 Factor Analysis

The results of factor analysis provide an interesting complement to evaluate the interdependence of the factors evaluated. In this respect, Figures 5–7 shows that the cutting time had a greater effect on the results than the morphological type of *L. aff*. *indica*. Whether in an individual manner or interacting with the other factor, the cut determined the differences obtained for all the variables evaluated. The cutting time affected the proximate composition, leaf quality, antioxidant properties, and chemical composition (macro- and microelements). In this regard, various researchers have published findings on the benefits of consecutive cutting for yield or formation of compounds in leafy vegetables, such as basil, lettuce, and arugula.^31,66,68,69^

The consecutive cutting technique has increased yield in leafy vegetables as it ensures earlier cutting, reduces production costs, and reduces labor costs by extending the crop cycle, thus avoiding multiply sowing times during the growing season.^66,68^ However, in this research, yield decreased as the cuttings progressed, going from 235.5 to 165.9 g plant^–1^ in morphological type SG, which was the most productive in the first cutting and the least productive in the third cutting, as discussed in the previous section, this decrease may be related to the type of cut, the number of days for regrowth, the ecophysiology of the plant,^65,67^ depletion of nutrients and energy reserves resulting from repeated cuttings, exhaustion of nutrient reserves, hormonal changes as a stress response, and reduction of the photosynthetic area.^70–72^

#### 4.2.1 Proximal Composition

Generally, high moisture percentages are common in leafy vegetables, also in the case of UFPs. This was shown by de Souza Ferreira et al.^73^ in their study on Barbados gooseberry (*Pereskia aculeata* Mill.), arrowleaf elephant ear (*Xanthosoma sagittifolium*), and vitória-régia (*Victoria amazonica*), in which moisture content was higher than 85%. Similarly, Costa et al.,^74^ evaluated UFPs biofortified with zinc, they found values around 86% of moisture in the leaves of *Lactuca spp*., when analyzed the control treatment, that is, without biofortification. Pimentel and Taiz et al.,^75,76^ state that of all the resources that plants need to grow and function, water is the most abundant and often the most restrictive, because water constitutes 80–95% of plant composition and is responsible for tissue turgidity, providing for good appearance; and this is ideal for sale of fresh leaves. As in these studies, the moisture content of *Lactuca aff. indica* leaves varied between 88.3 and 90.5%. However, these values were only influenced by the consecutive cutting and not by the variety, and the highest moisture content was found in the first cutting.

In proximate composition, the variety × cutting interaction was only significant for lipids, showing a significant increase in lipids between the first and third cuts, for SG and PP and from the first to the second cut in SP, with the maximum lipid value of 0.58% in the second SP cut. These lipid values are within the expected range according to Cecchi,^77^ who established values between 0.1 and 1.2% for plants. Lipids are highly energetic molecules, and they generally appear in low amounts in fruit and garden vegetables.^14^ In their study, they found lipid values of 0.53% in purple wild lettuce (without specifying whether it was plain or serrated), a value aligned with the values found in this study for the SG and PP varieties.

The other parameters related to proximate composition were determined only by the cutting. Thus, the highest values of ash, fiber, and caloric content were obtained in the first cutting, whereas the second cutting stood out for protein and carbohydrate levels. In this study, protein content ranged from 1.3 to 1.4% in the first and third cuttings, similar to the results of Silva et al.^26^ and exceeding the results of Silva et al.,^78^ who reported values of 1.32 and 1.25%, respectively, in the same species. However, in the second cutting, the species achieved levels of 1.7% protein, similar to the findings of Liberal et al.,^27^ who compared the species to a superfood, due to the 1.78% protein content found in its leaves, a value similar to that of *P. aculeata*.

Protein is an indispensable compound for growth, development, movement, heredity, and reproduction, among other activities of human life.^79^ Several authors have reported the advantages of replacing animal protein with plant protein.^80–82^ Therefore, due to the high protein content of *L. aff. indica* found in the second cutting, deeper studies should be conducted to be able to achieve these values over all the cuttings. The plant could be used to replace animal protein or to supplement the diet of people with low protein intake.

In the case of carbohydrates, the results varied depending on the cutting and on the varieties, with no interaction between these two factors. The second cutting had the highest percentage of carbohydrates (8.9%), while among the varieties, SG had the highest percentage (7.6%), though this was not significantly different for PP (7.2%). These values are higher than those found in other studies, which reported carbohydrate values of around 6.03–6.07% for *L. aff. indica*.^27,78^

Carbohydrate-rich foods of high quality are vital components of healthy dietary habits as they provide the body with glucose to support body functions and physical activity.^83,84^ The currently accepted positive carbohydrate quality indexes include plant-based foods, whole grains, and dietary fiber. In this context, *L. aff. indica* is an alternative to complement healthy diets that ensure intake of high-quality essential compounds. These carbohydrate values exceed those of other unconventional leafy vegetables such as Barbados gooseberry, lamb’s ear, arrowleaf elephant ear, sorrel, and nasturtium,^14,26^ making *L. aff. indica* a good option as a dietary supplement for carbohydrates.

The fiber content in the dry matter of the varieties of Wild lettuce evaluated was between 16,2% in the first and second cutting and 17.4% in the third cutting. These values are considered intermediate according to Silva et. al,^26^ who found similar values for the species. This fiber content in the leaves has health benefits, such as protection against type 2 diabetes, obesity, and cardiovascular diseases.^85^ The leaves of Wild lettuce are an interesting option for a healthy diet.

Ash content was also evaluated as part of the proximate composition. The values found exceeded 10% of the three cuts evaluated, with the first cutting standing out at 14.4% for the variety PP. However, these values are lower than those found by Liberal et al.,^27^ who reported ash values of 16.6% for this species. Ash content indicates inorganic substances in leaves, specifically significant amounts of minerals such as potassium, iron, calcium, phosphorus, magnesium, sulfur, and sodium.^86,87^ Therefore, considering that the highest ash content was obtained in the leaves in the first cutting, it can be assumed that their composition is dominated by nitrogen, phosphorus, potassium, and sulfur, which were the predominant minerals in that cutting.

Energetic value represents the energy provided by proteins, carbohydrates, and lipids^88^ present in leaf tissues. For *L. aff. indica* in this study, the energetic value was significantly higher in the second cut, with 4430 J. These values exceed those found by various researchers for Barbados gooseberry, arrow leaf elephant ear, Madeira vine, sow thistle, and sorrel,^14,89,90^ and certainly are consequence of the mineral contents, which will be discussed below.

#### 4.2.2 Physicochemical Parameters

Regarding the physicochemical parameter of the varieties of *L. aff. indica*, results suggest that it is affected more by the cutting than by the varieties. The first cutting had the lowest values of TTA (0;17%), TSS (2.94%), and the TSS/TTA (17.2), while pH was only significantly higher in the second cutting (6.3). However, all the values are within the ideal pH range for plant tissues suggested by Jay et al.^91^ Considering TSS, the first cutting had the lowest percentages (3%), while in the following cuttings, it was higher than 7.5%. According to Yommi et al.,^92^ the decrease in the TSS and TTA in young plants may be due to high sugar consumption during their rapid growth phase. This explains the significant increase in TSS and TTA in the leaves of the second cutting.

The TSS/TTA ratio is one of the indexes most used to determine the maturity and palatability of plant-based foods. It corresponds to the sugar and acidity content and is a suitable parameter for measuring consumer flavor perception.^93,94^ As a result for its individual parameters, the TSS/TTA ratio was significantly higher in the second cut. The first cutting had the lowest value, indicating its lower palatability. The flavor of fresh vegetables is due to the combination of volatile compounds with sugars and acids, which have a significant impact on sweetness perception, an effect on aroma, and an important effect on taste.^95,96^

The SPAD index decreased from the first to the second cut, but it showed significantly higher values (43.9) in the third cut (Figure 7F). According to El-Nakhel et al.,^68^ this increase is due to the successive cutting practice, and it may have arisen from improved photosynthetic efficiency.^66^

#### 4.2.3 Antioxidant Substances

The mechanical stress induced by successive cuttings can affect crucial leaf quality parameters, such as the bioactive compound profile.^97^ In their study on *Lactuca sativa* L., Flores et al.^69^ identified increases in total phenolic content after leaf cutting, attributing this response to the activation of physiological and biochemical mechanisms associated with the plant’s defense system. Among these responses is the increased synthesis and accumulation of total phenolic compounds. According to the authors, this occurs because the mechanical damage caused by cutting activates enzymes of the secondary metabolism, such as phenylalanine ammonia-lyase (PAL), which is involved in the phenylpropanoid pathway responsible for the biosynthesis of phenolic compounds. However, in the present study, an increase in total phenolic content was observed only from the first to the second cut in the PP variety (330.8 to 395.5 mg GAE 100 g^–1^). In the other evaluated conditions, the concentration of total phenolics showed a tendency toward significant reduction as the number of cuts progressed. In this context, it is important to consider that this condition may be influenced by several factors, such as variability among cultivars, which respond differently to the intensity and frequency of cutting due to their ecophysiological characteristics.^98^

In the case of AAc (Figure 5D), Their concentration was determined by the interaction variety: cut. Thus, its concentration increased from the first to the second cut, in the third cut, the concentration was significantly lower. The highest AAc concentration was 28.5 mg 100g-1 in the second SP cut. These values of ascorbic acid in the leaves are considered good by several authors, which favors the nutritional quality of the varieties.^30,99,100^ Similar ascorbic acid behavior was reported in a study evaluating *Chenopodium album* over three cuts.^30^

In addition, free radical scavenging increased significantly in the second and third cuttings. According to Flores et al.,^69^ successive cuttings play a crucial role in stimulating synthesis of phenolics, flavonoids, and anthocyanins; that is, in producing antioxidant compounds. Therefore, this strategy offers the possibility of producing leaves with different metabolic profiles and quality attributes.^97^

The presence of free radicals scavenging in this study was affected both by the cut and by the variety of Wild Lettuce, having the same behavior as ascorbic acid. Thus, the highest percentages were obtained in the second cut (17.2%). In the SG and SP varieties, FRS was significantly higher (17.4 and 14.0% respectively), which reduced oxidative stress and, consequently, the production of toxic compounds capable of affecting the organism, showing greater antioxidant capacity. Free radicals are highly unstable and reactive molecules formed in the body that participate in energy production and synthesis of biological substances.^101^ An excess of these radicals leads to oxidative stress, which attacks nearby molecules such as lipids, proteins, and fatty acids, altering their characteristics and producing toxic compounds that can result in degenerative diseases in the human body, such as aging, cancer, and Alzheimer’s disease.^89,102^ The results of this study are similar to the values of the antioxidant activity of asparagus (17.4%) and broccoli (11.5%) when determined by the same method (Sun et al., 2007).^124^

Among the antioxidant compounds capable of scavenging free radicals, phenolic compounds and ascorbic acid stand out. In this regard, Spagnol et al.; Tabart et al.,^101,103^ showed in their studies that phenolic compounds contribute much more to inhibition of oxidative damage than ascorbic acid does. Phenolic compounds act to eliminate reactive species, including free radicals, and continuously act in metal chelation in the initiation phase and in oxidative processes.^104^ Ascorbic acid carries out important functions in the body, such as formation of cartilage, collagen, muscles, and blood veins.^105^ It is a powerful antioxidant and can protect molecules such as proteins, carbohydrates, nucleic acids, and lipids from damage caused by free radicals.

Some studies have shown a relationship between an antioxidant-rich diet and protection against diseases resulting from oxidative damage, leading to growing interest in a healthy diet rich in antioxidants.^69,89,103,106^ The determination of the antioxidant capacity of foods should consider the concentrations and overall compositions of various antioxidants, because total antioxidant capacity is due to the combination of these compounds.^103^

In this respect, as it concentrates phenolic compounds and ascorbic acid (Figure 5C,D), *L. aff. indica* emerges as an alternative food with antioxidant properties. The first cut had a higher concentration of TP for SG and SP; while in the second cut of leaves, the TP concentration was significantly higher for PP. These results differ from those found for different types of lettuce, where an increase in phenolics was observed from the first to the third cut.^69^ Ascorbic acid, in turn, showed the highest values in the second cut for all varieties, in the following order: SG > SP > PP. Considering the antioxidants evaluated and the FRS, the greater scavenging of free radicals in the second cut may primarily be affected by vitamin C. Furthermore, the SG phenotype has the best antioxidant properties.

#### 4.2.4 Macro and Microelements

When analyzing mineral nutrition, we must consider that humans consistently require at least 51 known nutrients in adequate amounts to live healthy and productive lives.^107^ Therefore, it is important to ensure consumption of healthy foods that provide these nutrients. High mineral content is one of the characteristics that has led to increased interest in UFPs,^88^ due to their ability to promote health and prevent and treat pathologies.^35^ The percentage of proteins and the ash content, as previously discussed, can indicate the mineral content in the edible leaves of vegetables.^27^ In their study, working with *L. aff. Canadenses*, Botrel et al.^14^ found 533.5 mg·100 g^–1^ of potassium in the leaves of Wild lettuce, a value higher than the values found in this study, where the highest concentration occurred in the first cutting (305 mg g^–1^). The same authors showed lower values for other elements, such as half the levels of Ca, Mg, and Fe, and less than a third the levels of Mn and Cu. In contrast, a study performed with *Pereskia aculiata*, a UFP recognized as superfood. Ferreira et al.,^108^ showed values for the minerals Ca, Fe, and Zn near those found here for *L. aff. indica*. Moreover, the SG and SP varieties doubled the P value reported for that species.

Of the mineral nutrients evaluated in the chemical composition, only N and P accumulation was affected by the morphological type × cutting interaction. The SG × 1 interaction led to the highest concentration of N (in mg g^–1^) in the leaves, while the SP × 1 interaction produced the greatest accumulation of P in the leaves (Figure 6A,B). N plays an integral role in plant nutrition, is involved in photosynthesis, and is a key component of proteins, amino acids, nucleic acids, enzymes, hormones, and chlorophyll,^109–111^ directly affecting the nutritional quality of the leaves. Additionally, all the main quality attributes in vegetable crops, including visual quality and flavor, are directly affected by N availability.^112^ Figure 6A shows that SG and SP stood out for higher N levels in the first cutting (330 mg·100 g^–1^). However, these values decreased as the cuttings advanced, with a minimum of 165 mg·100 g^–1^ for SP in the third cutting. The nitrogen values found in this study differ from the results of dos Santos Viana et al.,^113^ who found values greater than 400 mg·100 g^–1^; and they stated that ideal N levels in Wild lettuce are from 400 to 500 mg·100 g^–1^.

In leafy vegetables, the relationship between the yield and biogenic elements is generally analyzed regarding nitrogen.^97^ According to this principle, in our study, the SG variety of *L. aff. indica* was the most nutrient-rich in the first two cuttings, while PP was the type with the highest nutrient content in the third cutting (Figure 6A). Furthermore, the positive correlation between N and moisture found in the SOM (Figure 6B) can be explained based on the results of Qiu et al.^114^—in their study on leafy vegetables (rape seed, bok choy, and spinach), they showed a positive linear correlation between nitrate concentrations and moisture content in the plant tissues. Similarly, Burns et al.,^115,116^ working with a large number of *L. sativa* *L* and *Lactuca serriola* *L* accessions, found that water accumulation is a plant strategy for diluting nitrate, helping to regulate its concentrations.

Phosphorus is a nutrient essential for human life and it performs vital functions in skeletal and nonskeletal tissues. It is fundamental for energy production, acts as a signaling molecule, and induces complex physiological responses.^117^ However, some authors recommend avoiding consumption of phosphorus-containing additives used in food manufacture and processing, as excessive intake of this nutrient could damage health, especially the renal and endocrine systems.^117,118^ These authors recommend consumption of natural foods to ensure P intake, as in legumes, dairy products, eggs, and vegetables. As a vegetable, *L. aff. indica* represents an excellent alternative, for according to our results, it ensures intake of 35.5 mg (SG variety, first cutting) and 36.1 mg (SP, first cutting) of P per 100 g^–1^ of fresh leaves consumed. These values exceed those of other UFPs, such as Barbados gooseberry, lamb’s ear, spilanthes, and amaranth greens.^14^

The accumulation of other minerals in the leaves according to the varieties of *L. aff. indica* evaluated was significantly different only for Ca, Mg, S, and Mn (Figure 7F,I). The SG variety had the highest concentration of these minerals, except for Mg, which was highest in PP. The other minerals evaluated showed variation depending on the cutting. Thus, enrichment was observed in the second cutting; and this was maintained for Ca, Mg, Cu, Fe, Mn, and Zn up to the third cutting (Figure 7O,P,R–T,V).

The micronutrients evaluated in the leaves of wild lettuce have vital importance in the diet of the consumer. However, Assunção et al.,^119^ emphasize their importance for N metabolism, as well as their interaction with each other and with various macronutrients. Therefore, they also indirectly affect the formation of proteins and amino acids. Micronutrients such as Zn, Cu, and Fe are necessary for various critical functions, including cognition, development, immune response, immune system, cholesterol, hematological control, maintenance of antioxidant activity, and mitigation of chronic diseases.^120–122^ The values obtained in this study for these three micronutrients in the second and third cuttings were greater than those obtained for purple Wild lettuce in a single cutting.^14^

These results of the factorial analysis indicate that Wild lettuce leaves have antioxidant properties and nutritional quality comparable to leafy vegetable species considered superfoods. In other hand, green Wild lettuce (SG) stood out from the other varieties, and the nutritional quality of the plants was substantially better in the third cut, suggesting that choosing the ideal cutting time will increase producer profits and allow growers to send higher-quality vegetables to the market.

When comparing the results of the factor analysis with those obtained through PCA and SOM, it is possible to observe the similarity among them, demonstrating that all these methods can be suitable for analyzing data related to chemical composition, nutritional quality, and plant yields, with the aim of separating varieties based on these characteristics. However, considering that our focus was to test a method such as SOM, which is still rarely applied in the characterization and differentiation of plant varieties and is based on neural networks and machine learning, in comparison with the most commonly used multivariate methods, we believe that our objective was achieved. The SOM method proved to be efficient in data analysis and particularly valuable in the presentation of the results, allowing us to recommend the use of this methodology for the separation and differentiation of plant varieties. and more specifically of nonconventional food plants, to generate useful information for human nutrition.

## 5 Conclusions

The validation of the multivariate analyses applied in this study demonstrates their suitability for data analysis. However, the Self-Organizing Map (SOM) enabled a more efficient examination of the clustering tendency among treatments, facilitating pattern recognition and allowing for a clearer interpretation of the data based on the proximity and similarity of colors and positions. Thus, the data were grouped into seven clearly defined clusters, depending on the variables that most influence each treatment.

Wild lettuce yield varied depending on the harvest time; it was highest in the first cut (225.1 g plant^–1^) and tended to decrease over consecutive cutting (171.0 g plant^–1^).

Results indicate that Wild lettuce leaves have antioxidant properties and physicochemical and nutritional quality comparable to leafy vegetable species considered superfoods.

Serrated green Wild lettuce (SG) stood out from the other varieties, and the nutritional quality of the plants was substantially highest when the leaves were cut 35 days after the first cutting, suggesting that choosing the ideal harvest time will increase producer profits and allow growers to send higher-quality vegetables to the market.
